# Supplementary material for: Isotopologue-induced structural dynamics of a triazolate metal-organic framework for efficient hydrogen isotope separation
Source: Nat Commun. 2025 Jul 1;16:5941. doi: 10.1038/s41467-025-61107-3 (PMC12215783; doi:10.1038/s41467-025-61107-3)
Supplement: Supplementary file 1 — Supplementary Information [file 41467_2025_61107_MOESM1_ESM.pdf]

Supplementary Information

Isotopologue-Induced Structural Dynamics of a Triazolate Metal-Organic Framework for Efficient Hydrogen Isotope Separation

Linda Zhang[1,2*], Richard Röß-Ohlenroth[3], Vanessa K. Peterson[4], Samuel Duyker[5], Cheng Li[6], Jhonatan Luiz Fiorio[7], Jan-Ole Joswig[7], Robert Dinnebier[8], Dirk Volkmer[3], Michael Hirscher[2,9*]

[1] Frontier Research Institute for Interdisciplinary Sciences, Tohoku University
6-3 Aramaki-Aoba, Aoba-ku, Sendai, 980-0845, Japan

[2] Advanced Institute for Materials Research (WPI-AIMR), Tohoku University
2-1-1 Katahira, Aoba-ku, Sendai, 980-8577 Japan

[3] Chair of Solid State and Materials Chemistry, Institute of Physics, University of Augsburg
Universitätsstraße 1, 86159 Augsburg, Germany

[4] Australian Centre for Neutron Scattering, Australian Nuclear Science and Technology Organisation
New Illawarra Road, Lucas Heights, NSW 2234, Australia

[5] Sydney Analytical, The University of Sydney
Locked Bag 2001, Kirrawee DC 2232, Australia

[6] Spallation Neutron Source, Oak Ridge National Laboratory
1 Bethel Valley Road, Oak Ridge, TN 37830, United States

[7] Theoretische Chemie, Technische Universität Dresden
Bergstraße 66c, 01069 Dresden, Germany

[8] Max Planck Institute for Solid State Research
Heisenbergstraße 1, 70569 Stuttgart, Germany

[9] Max Planck Institute for Intelligent Systems
Heisenbergstraße 3, 70569 Stuttgart, Germany

E-mail: linda.zhang.a3@tohoku.ac.jp; hirscher@is.mpg.de

Structure Analysis and Characterization

XRPD data

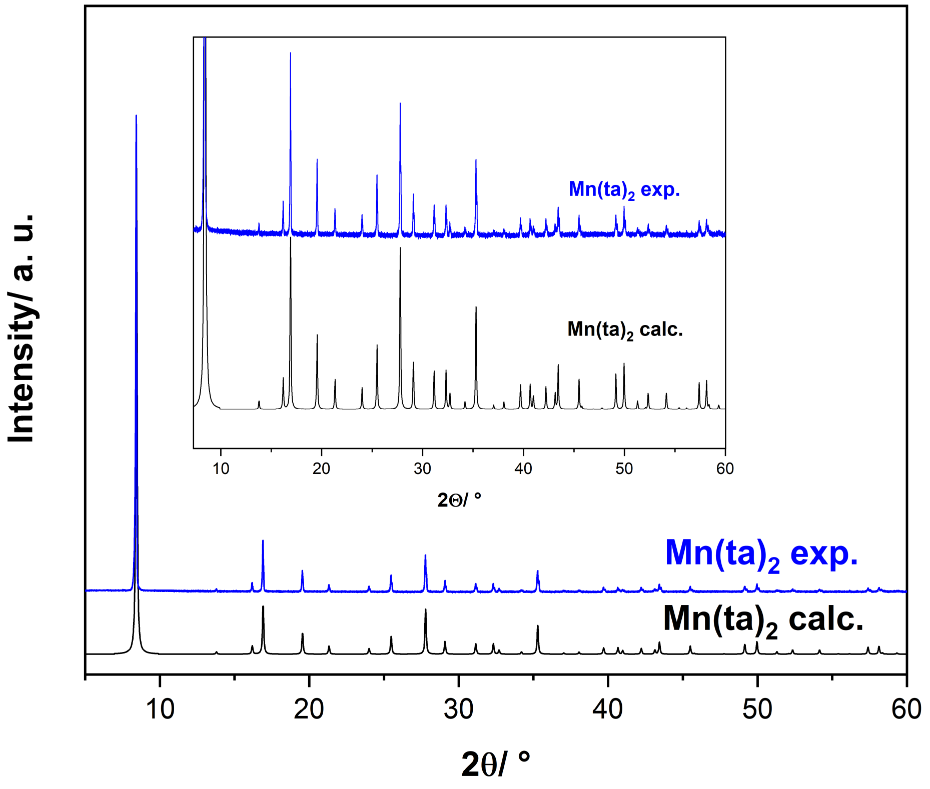

Supplementary Figure 1. **Structure Mn(ta)$_2$.** XRPD data calculated (calc.) from the literature [1] and measured (exp.) for Mn(ta)$_2$. Data are shown as a line and offset in y for clarity.

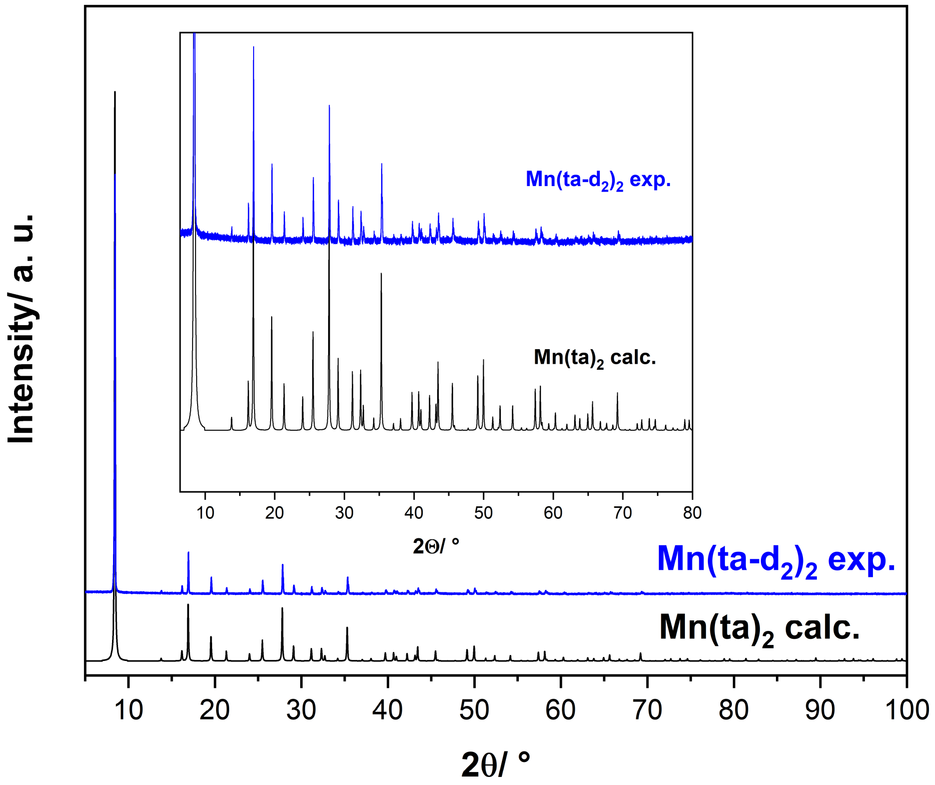

Supplementary Figure 2. **Structure Mn(ta-d$_2$)$_2$.** XRPD data calculated (calc.) from the literature [2] for Mn(ta)$_2$ and measured (exp.) for Mn(ta-d$_2$)$_2$. Data are shown as a line and offset in y for clarity.

SEM micrographs

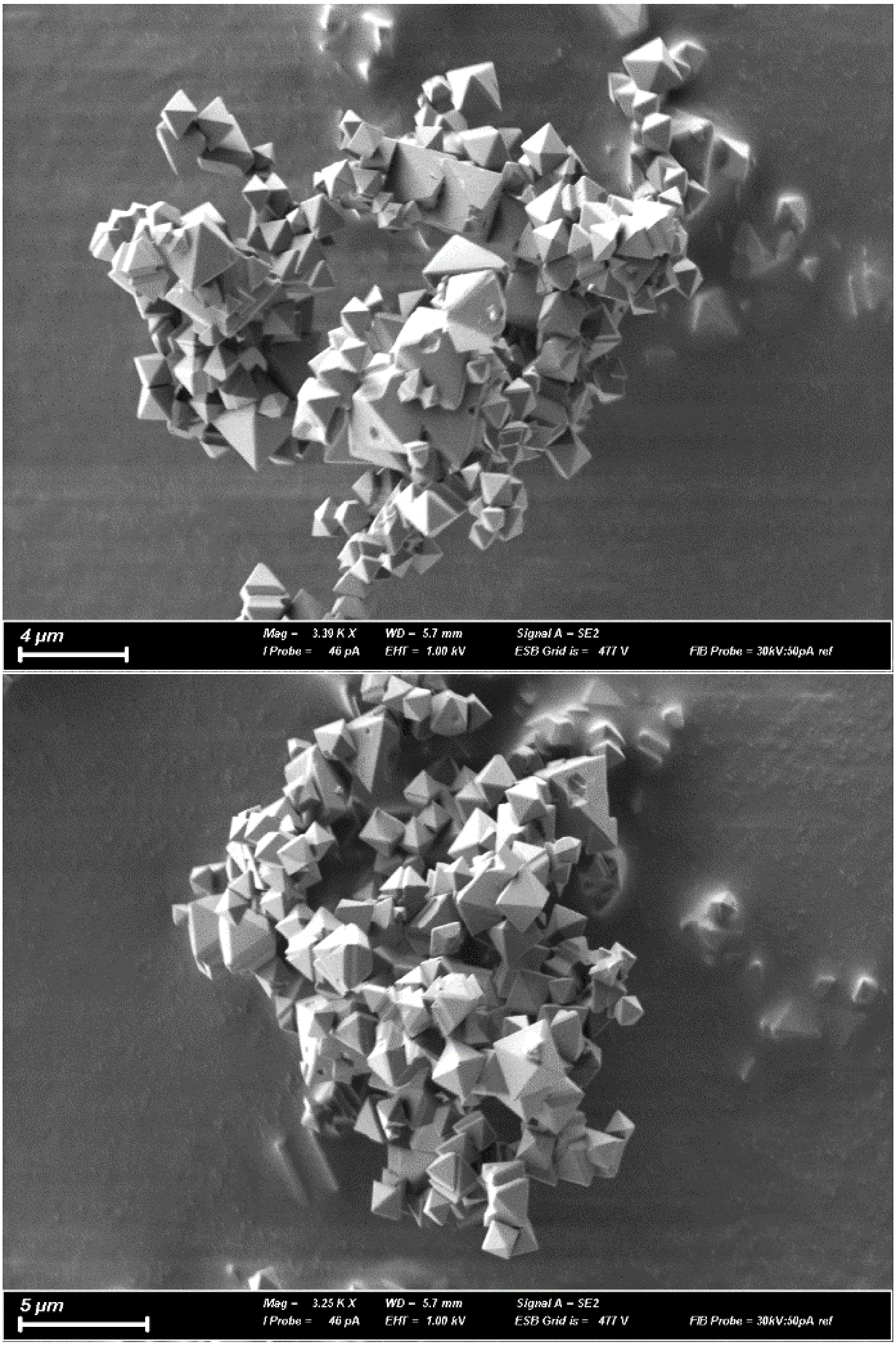

Supplementary Figure 3. **SEM micrographs of $Mn(ta)_2$**

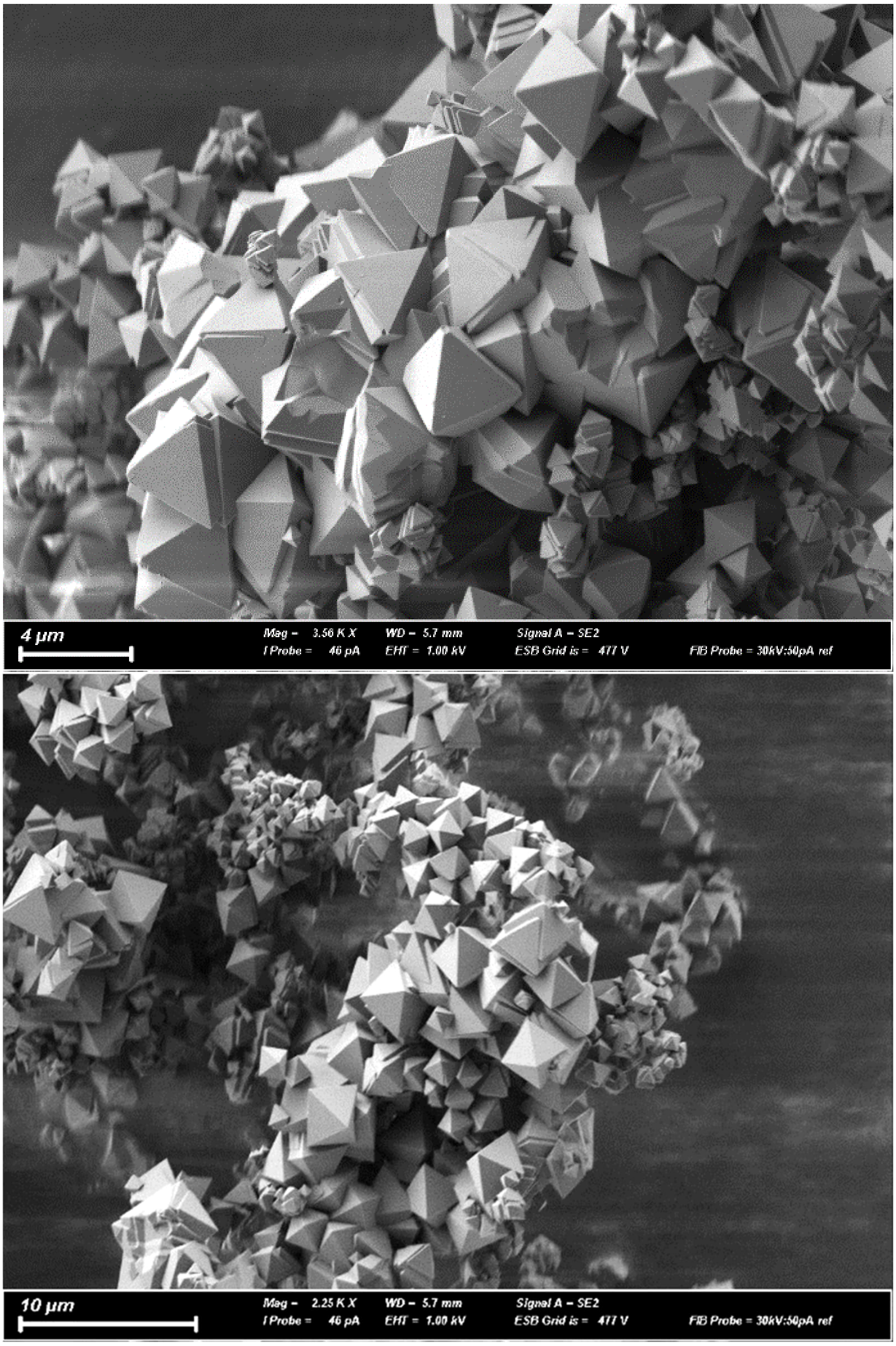

Supplementary Figure 4. **SEM micrographs of $Mn(ta\text{-}d_2)_2$**

FT-IR spectra

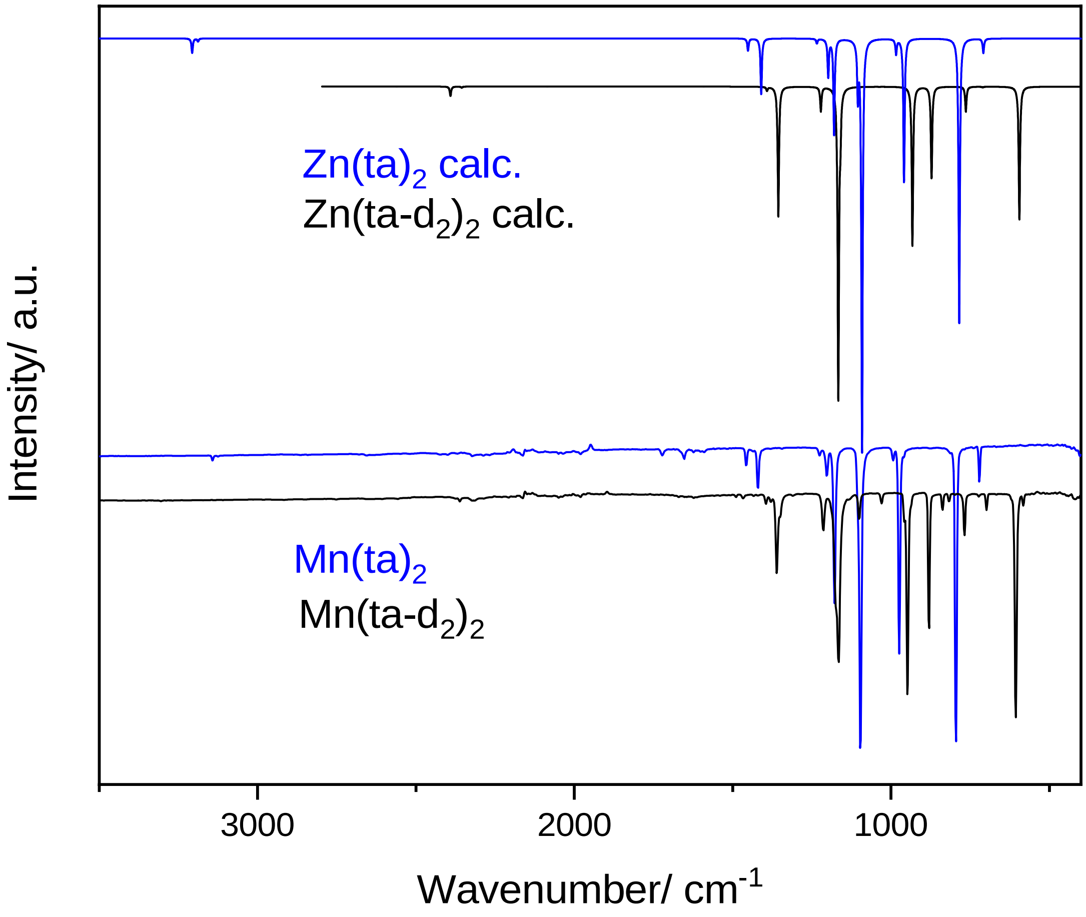

Supplementary Figure 5**. Overview FT-IR spectra.** Comparison of simulated (Zn derivatives) and measured FT-IR spectra of $Mn(ta)_2$ and $Mn(ta\text{-}d_2)_2$ showing the differences between both samples. For $Mn(ta)_2$ FT-IR (ATR) 4000–400 cm–1: 3143 (w), 1722 (w), 1653 (w), 1457 (w), 1420 (w), 1226 (w), 1202 (w), 1178 (m), 1096 (s), 993 (w), 974 (m), 794 (s), 721 (w). For $Mn(ta\text{-}d_2)_2$ FT-IR (ATR) 4000–400 cm–1: 2362 (vw), 1623 (w), 1395 (w), 1361 (m), 1214 (w), 1165 (s), 1100 (w), 1030 (w), 948 (s), 880 (m), 837 (w), 817 (w), 768 (w), 698 (w), 606 (w), 582 (w)

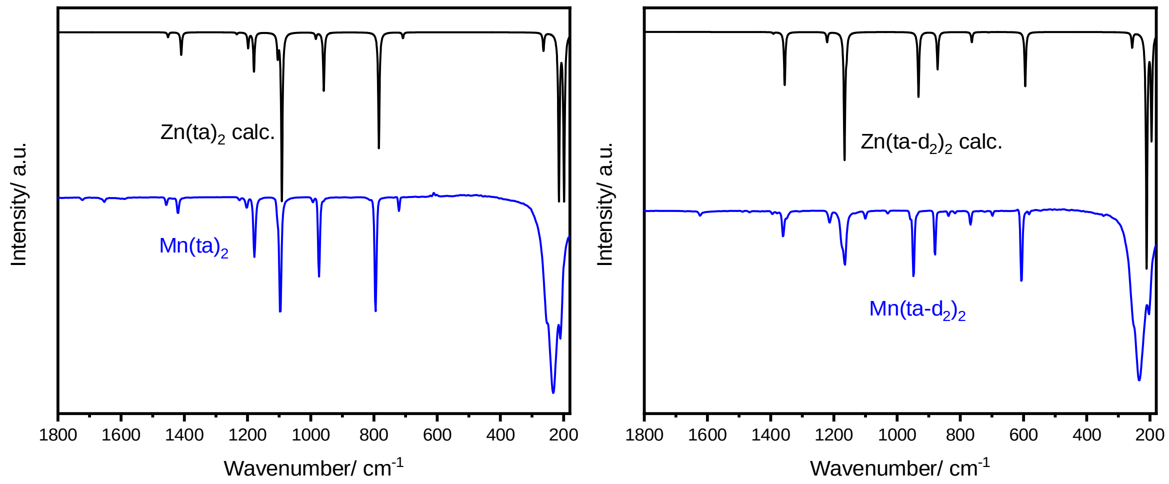

Supplementary Figure 6. **Direct comparison at low wavenumbers.** Simulated (Zn derivatives) and measured FT-IR spectra in the range of 1800-180 cm^{-1} of $Mn(ta)_2$ and $Mn(ta\text{-}d_2)_2$ showing a good match.

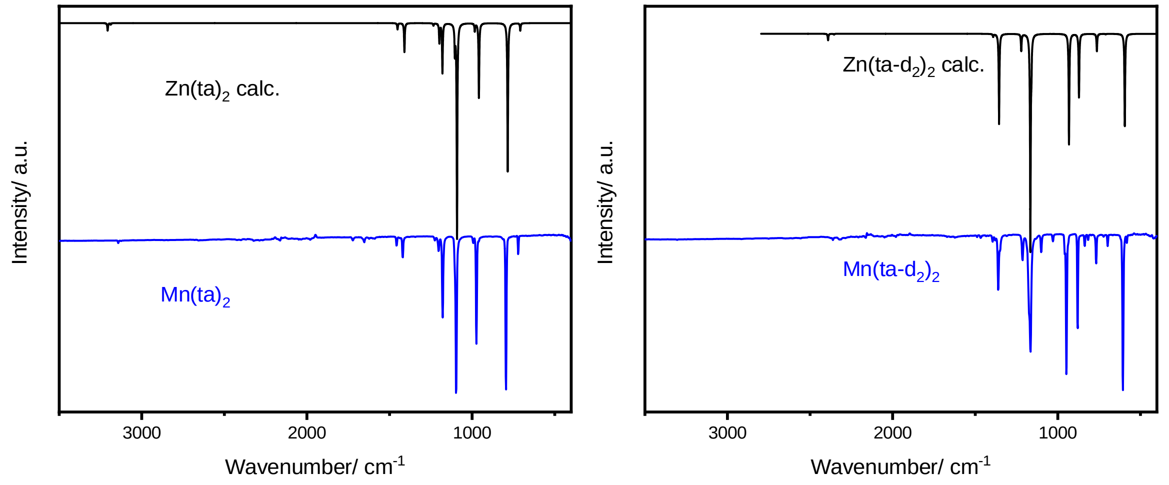

Supplementary Figure 7. **Direct comparison at high wavenumbers.** Simulated (Zn derivatives) and measured FT-IR spectra in the range of 4000-400 cm^{-1} of $Mn(ta)_2$ and $Mn(ta\text{-}d_2)_2$ showing a good match.

TGA data

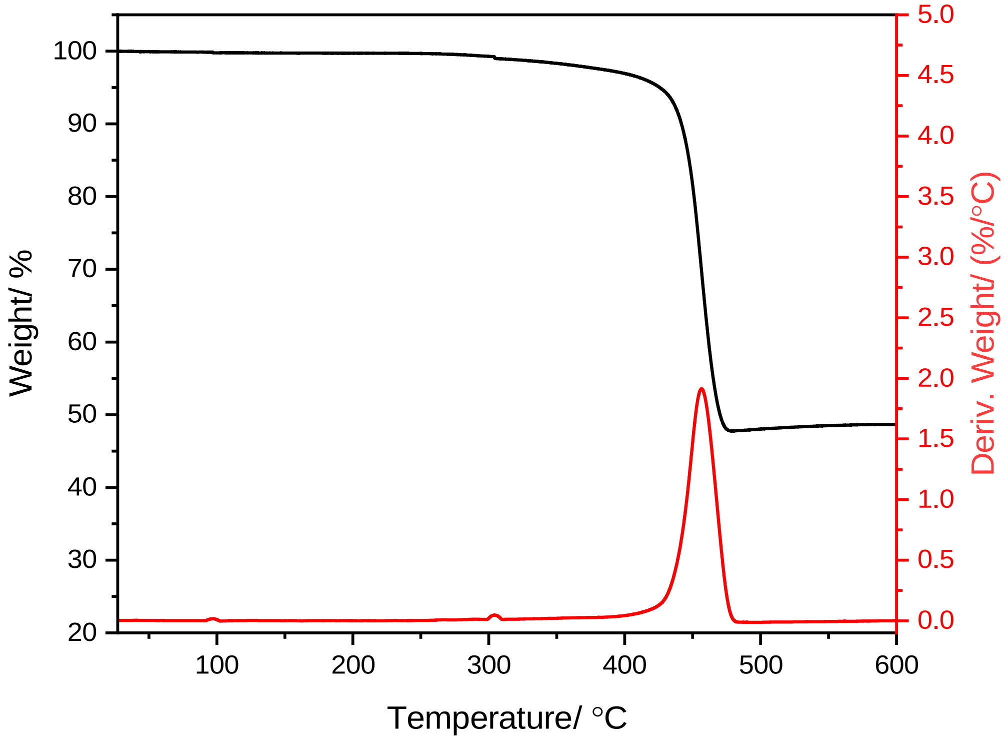

Supplementary Figure 8. **Thermal gravimetric analysis.** TGA data of $Mn(ta\text{-}d_2)_2$

Gas sorption isotherms

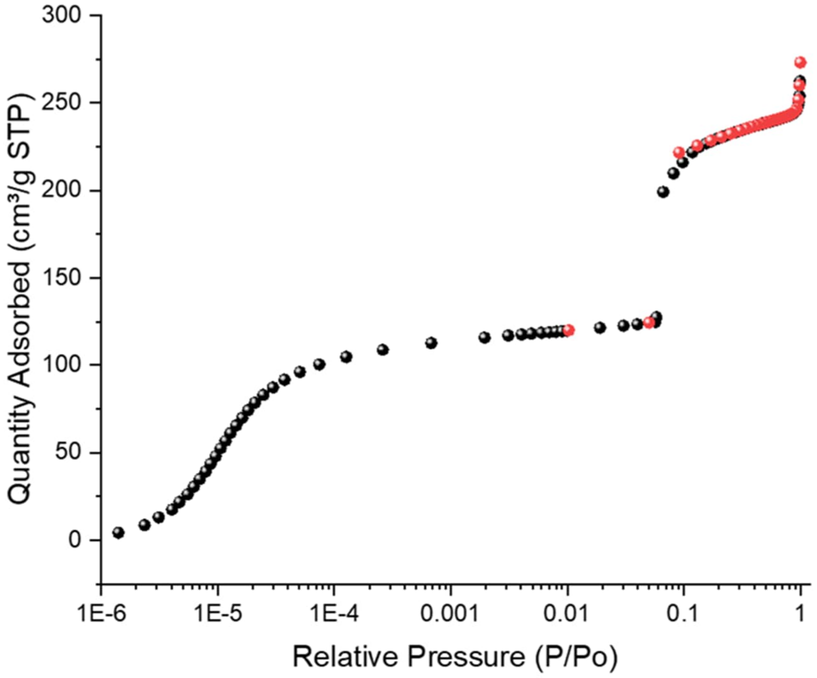

Supplementary Figure 9. **Nitrogen (N_2) sorption.** Isotherms at 77 K for $Mn(ta)_2$. Solid symbols: adsorption; hollow symbols: desorption.

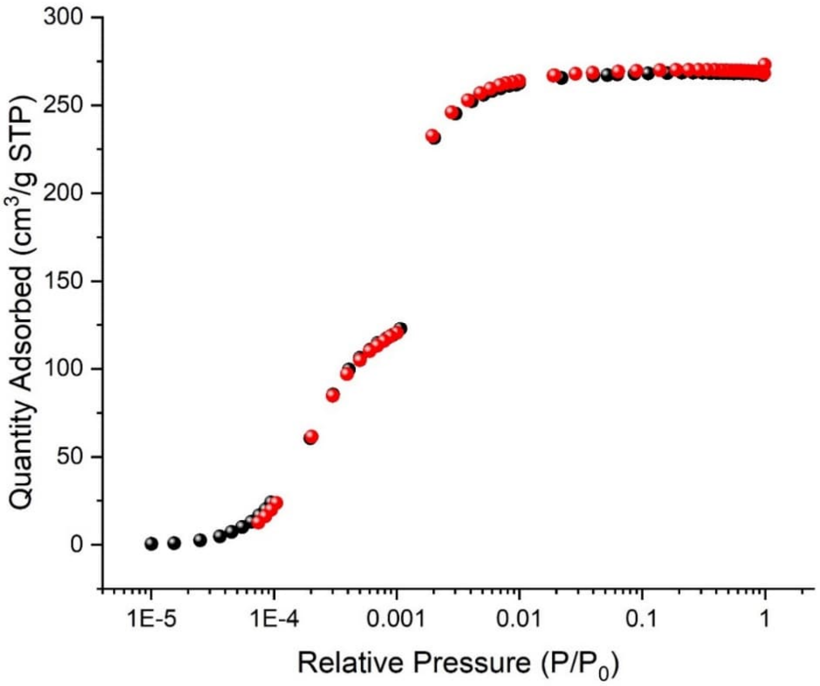

Supplementary Figure 10. **Argon (Ar) sorption.** Isotherms at 87 K for $Mn(ta)_2$. Solid symbols: adsorption; hollow symbols: desorption.

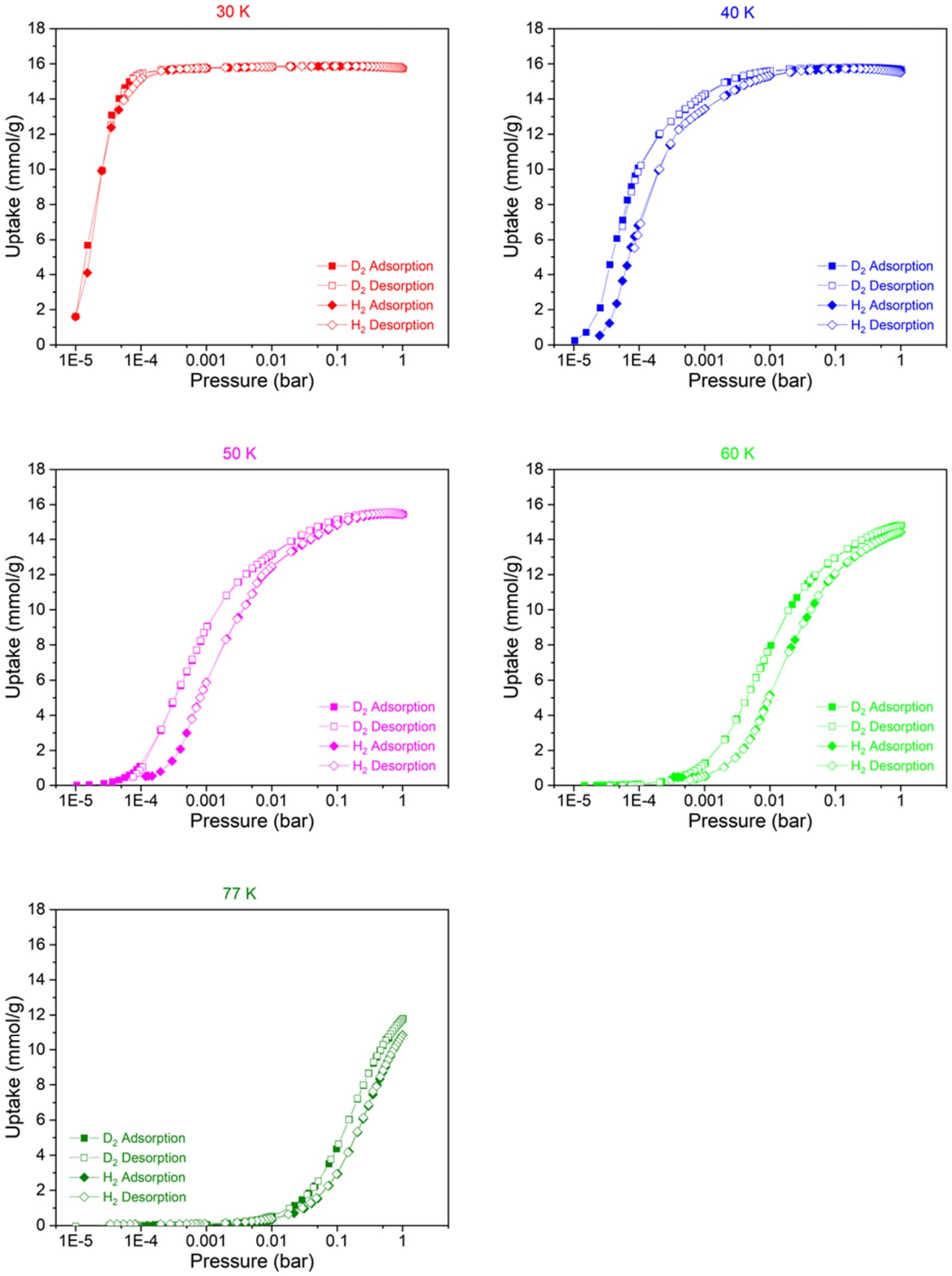

Supplementary Figure 11. **Direct comparison H_2 and D_2 gas sorption.** Single-component isotherms collected at 30 K, 40 K, 50 K, 60 K, and 77 K, respectively.

In Situ Neutron Powder Diffraction and Analysis

Typical refinement parameters for NPD data analysis

Instrumental and Refinement Parameters

Refinement of the empty MOF structure was carried out using the Rietveld method. The background was modeled using a Chebyshev polynomial of order 10. The following instrumental parameters were used:

- Wavelength: 1.622067 Å (delta function)
- Axial divergence correction: Simple axial model, 37.58(15) mm
- Convolution function: Hat profile with a constant of 0.32(4)
- Zero-point error: −0.0607(12)
- Lorentz–polarization (LP) factor: 90
- Convolution steps: 2
- X calculation step size: 0.02
- X-range: 6 to 155

Supplementary Table 1: Background: Chebyshev Polynomial coefficients

Coefficient No.	Value
0	2304(8)
1	-789(10)
2	597(8)
3	-409(7)
4	358(7)
5	-198(6)
6	226(6)
7	-99(6)
8	105(5)
9	-61(5)
10	24(4)

Unit cell and refinement summary

- Space group: Fd-3m:2
- R-Bragg: 1.917
- Scale factor: 0.00680(3)
- Cell mass: 4585.267
- Cell volume: 5965.73(15) Å^3
- Lattice parameter a: 18.13654(15) Å

• Peak shape function: PV_TCHZ

U	0.097(2)
V	−0.375(6)
W	0.430(14)
X	0
Y	0.060(3)
Z	0

Supplementary Table 2. Atomic coordinates and displacement parameters for the empty MOF

Site	Np	x	y	z	Atom	Occupancy	Beq
Zn1	16	0.00000	0.50000	0.00000	Mn	1	0.00(8)
Zn2	8	0.87500	0.37500	-0.12500	Mn	1	0.00(11)
N1	96	0.91763(4)	0.41763(4)	0.04202(5)	N	1	0.376(19)
N2	48	0.87500	0.37500	0.00015(9)	N	1	0.46(2)
C1	96	0.90178(5)	0.40178(5)	0.11265(8)	C	1	0.49(2)
D1	96	0.92964(7)	0.42964(7)	0.15741(9)	D	1	1.88(3)

Neutron powder diffraction data

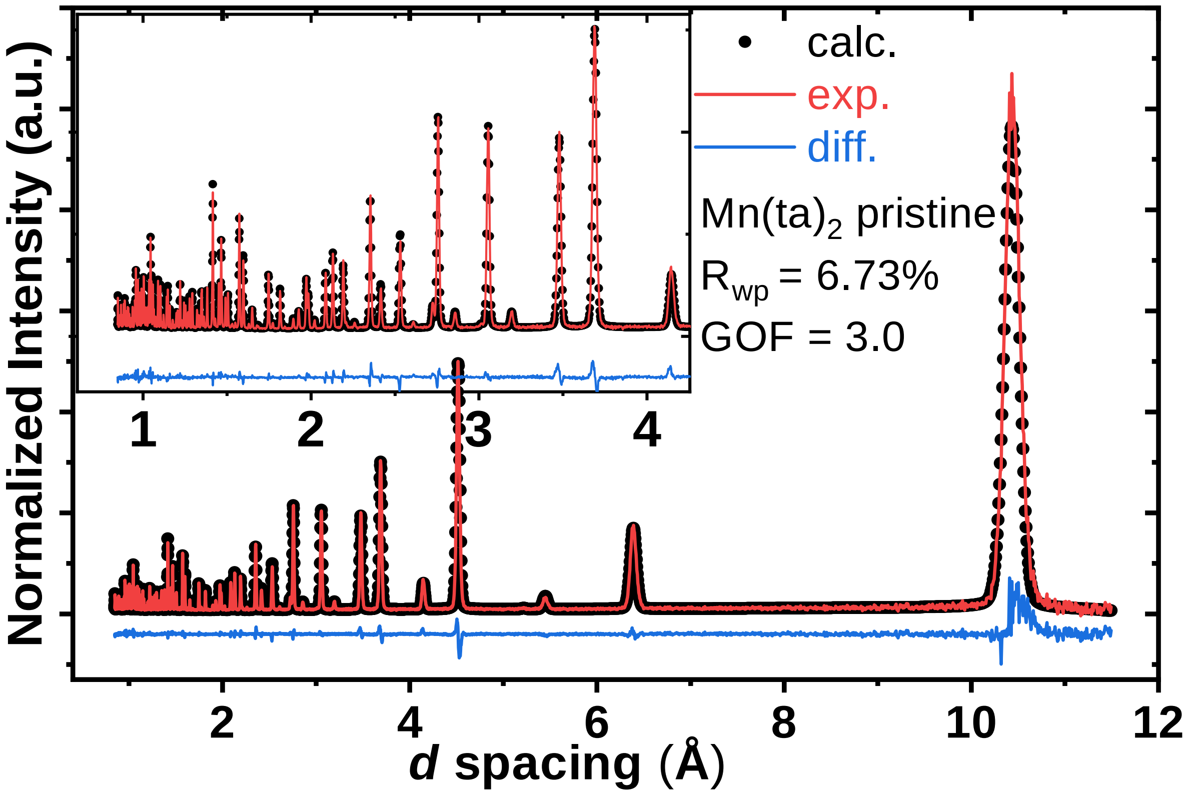

Supplementary Figure 12. **NPD Rietveld refinement profile pristine sample.** As prepared $Mn(ta\text{-}d_2)_2$ at 15 K using NPD data. Figures of merit are the goodness of fit (GOF) and the weighted profile R-factor (R_{wp}).

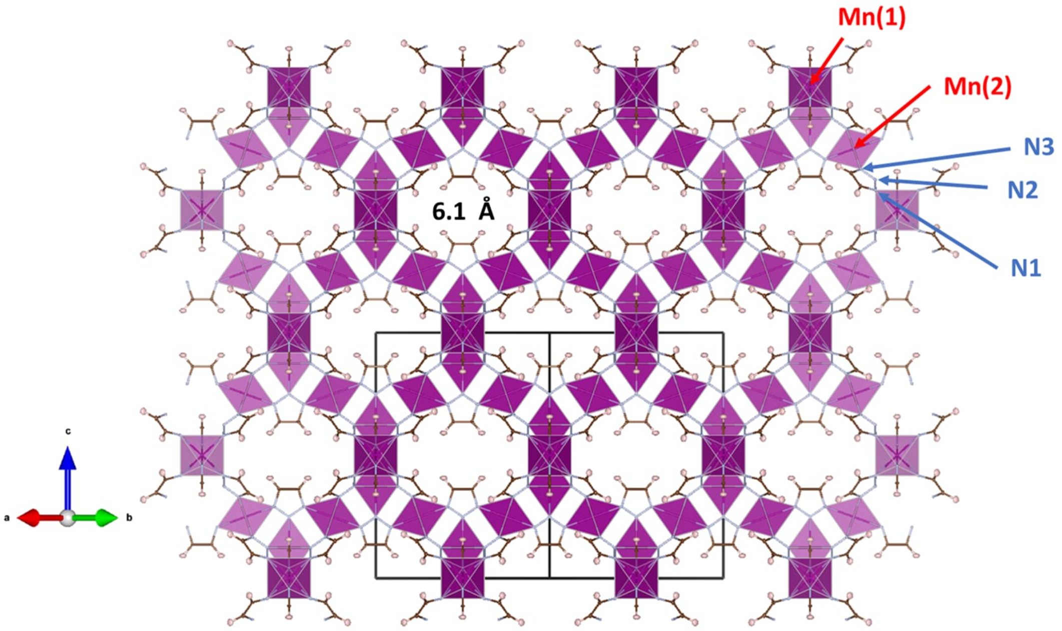

Supplementary Figure 13. **Structure of [Mn(ta-d$_2$)$_2$].** Refinement using NPD data viewed along the [110]. Magenta: Mn, Blue: N, Brown: C, Light red: H. The sample contains cavities with a diameter of ~ 6.1 Å and the empty volume accounts for ~ 40% of the space in the framework.

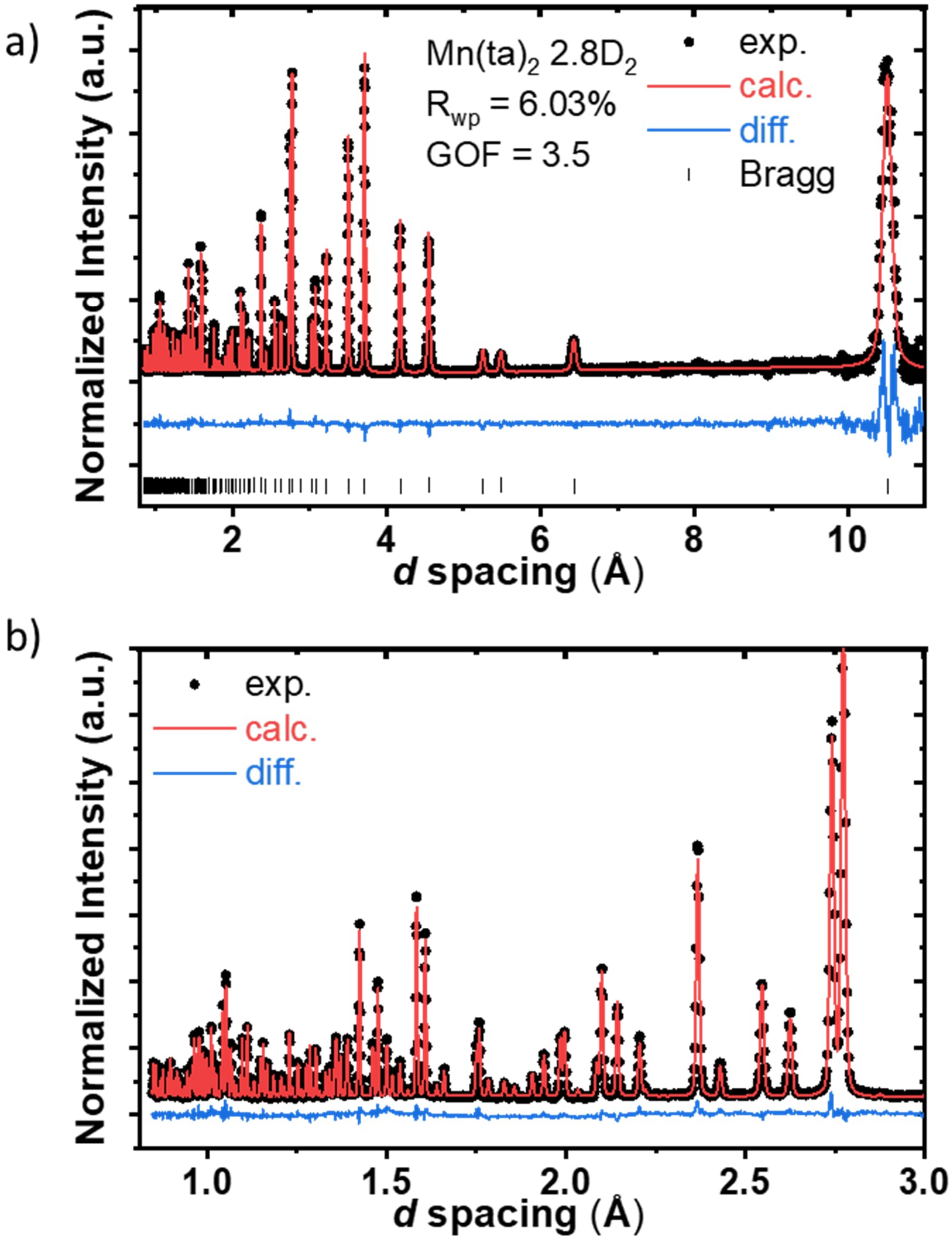

Supplementary Figure 14. **NPD Rietveld refinement profile D_2 loaded sample.** Refinement profile using NPD data of (a) Mn(ta-d_2)$_2$ with 2.8 D_2 (15 mmol/g D_2) at 15 K; (b) Low-d section of (a). Figures of merit are the goodness of fit (GOF) and the weighted profile R-factor (R_{wp}).

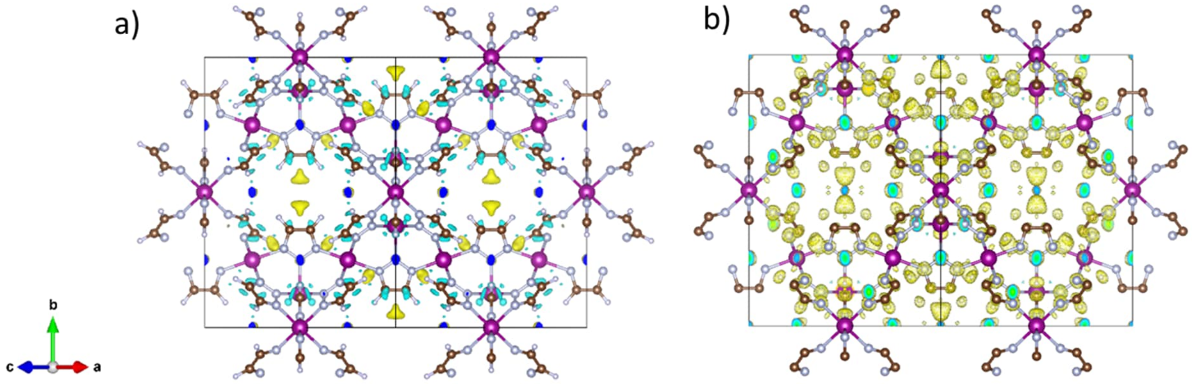

Supplementary Figure 15. **Fourier difference map of isotope loaded sample**. NPD data of [Mn(ta-d$_2$)$_2$] loaded with 15 mmol/g D_2 or H_2 showing the refined framework structure and nuclear density arising from (a) H_2 and (b) D_2 in the framework (yellow with a dark blue intersection of unit cell faces.

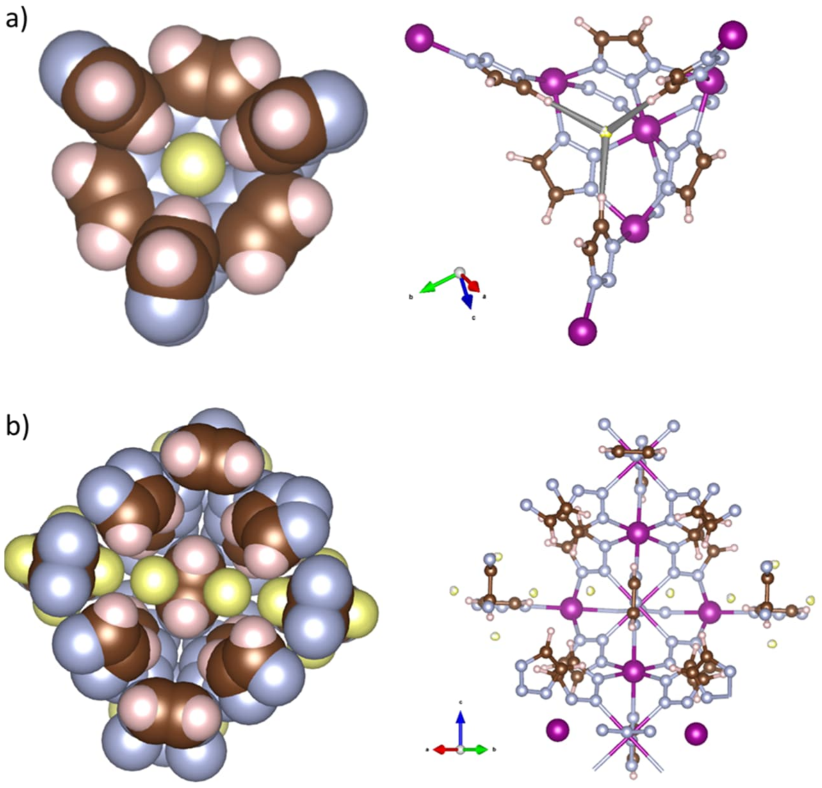

Supplementary Figure 16. **H_2 adsorption sties 1 and 2.** a) Schematic of the short-range structure around adsorption Site I, located inside the entrance of the pocket created by the ta-ligands of [Mn(ta)$_2$], from [111] view; and b) adsorption Site 2, along [001] view, located in the pore channel. Magenta: Mn, Blue: N, Brown: C, Pale Red: D. Adsorbed H_2 are represented in yellow.

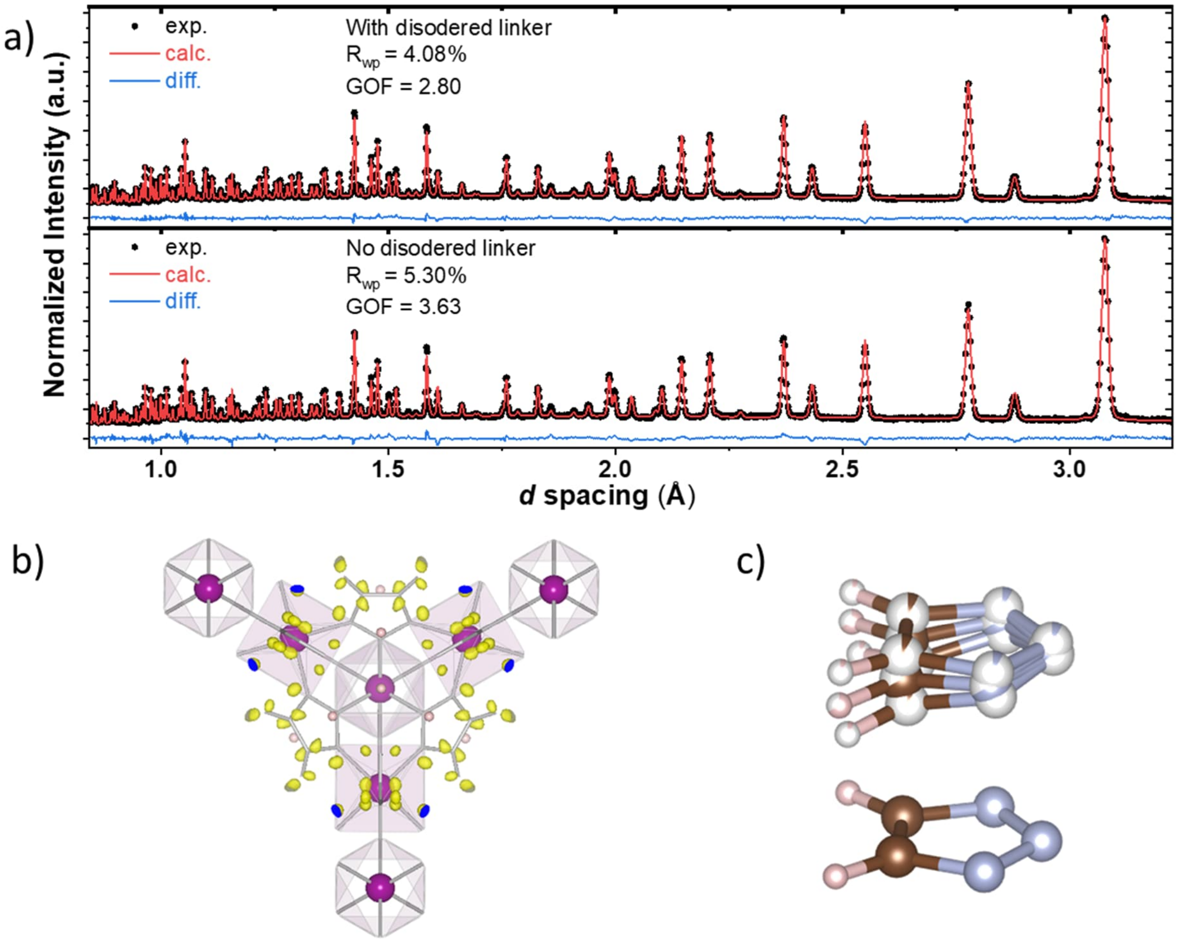

Supplementary Figure 17**. NPD H_2 loaded sample.** a) Rietveld refinement profile using NPD data of Mn(ta-d_2)$_2$ with 2.4 mmol/g H_2 measured at 6 K using a structural model with and without a disordered linker. The motel with disordered linker produces noticeable improvement in the fitting quality. Figures of merit are the goodness of fit (GOF) and the weighted profile R-factor (R_{wp}); b) Fourier difference map obtained from the refinement using the disordered linker model showing the positive residual scattering length density (yellow with a dark blue intersection of unit cell faces) that is perpendicular to the linker group plane; isosurface level = 0.03 fm A^{-3}; F_{000} = 0.66 fm A^{-3}; c) schematic showing the normal and disordered linker configuration, brown: carbon, blue: nitrogen, pink: deuterium.

Supplementary Table 3. **Location of H_2 and D_2.** Distance (Å) between the D_2 and H_2 adsorption sites of [Mn(ta)$_2$] and their neighbors*

	ads. site 1 - D_{linker}	ads. site 1 - N2	ads. site 1 - D_{link}	ads. site2 – ads. site2
D_2	2.494(4)	3.184(3)	3.858(4)	3.668(6)
H_2	2.475(5)	3.202(4)	3.841(9)	3.634(13)

* The distance was reported based on the refinement shown in Figure 3(a) & 3(b) and Figure S13 for the H_2 and D_2 loaded Mn(ta)$_2$ respectively. The location of the adsorption site 1 and 2 are shown schematically in Figure 3c. Additional information about the structure could be found in the CIF files in the SI. Distances are reported for assuming D_2 adsorption at 32*e* sites.

Density Functional Calculations

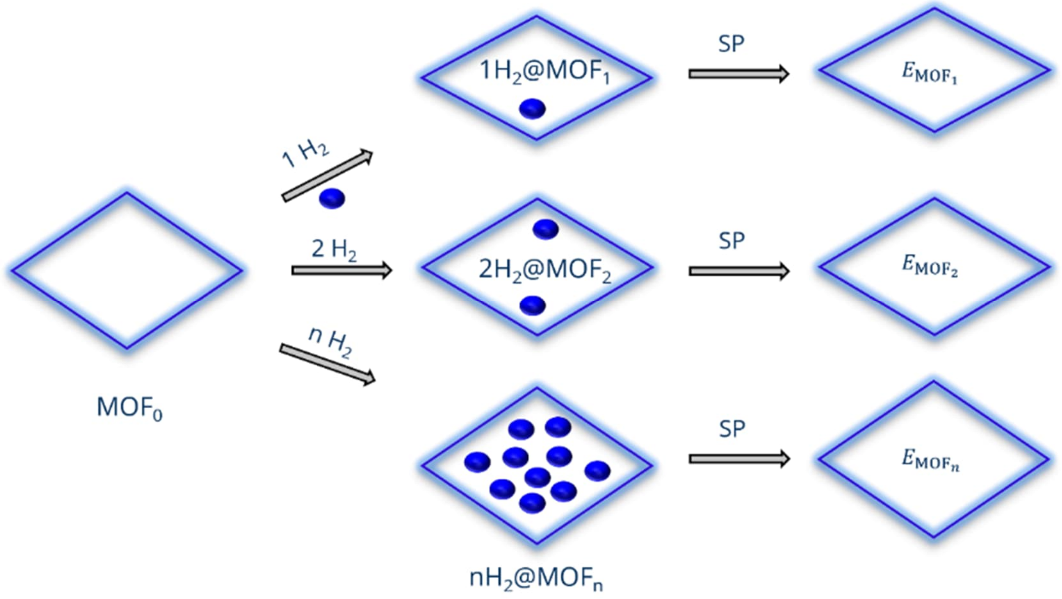

Supplementary Figure 18. **Schematic illustration of the calculational procedure and respective nomenclature.** The framework (blue lines) is infiltrated with the H_2 molecules (blue spheres) and fully relaxed to assess the energetic contribution of adding guest molecules. The index n in MOF_n denotes the MOF structure in an opening to incorporate n H_2 molecules (fully lattice and geometry-optimized). It might be used in a filled context, e.g. as $2H_2@MOF_2$, or in an empty context, e.g. as MOF_2. Energies are labeled accordingly: Thus, $E(nH_2@MOF_n)$ denotes the total energy of a MOF loaded with n H_2 molecules, and $E(MOF_n)$ is the total energy of the corresponding emptied MOF structures resulting from a single-point calculation.

Supplementary Table 4. Unit-cell volume, lattice parameters, and selected bond lengths of $[Mn(ta)_2]$ obtained by DFT calculations s described above alongside with reported experimental values.

**Quantity**		**DFT (this work)**	**Experiment [reference]**
**Unit-cell volume [Å^3]**		5991.8	5971.7 [1]
**Lattice parameter [Å]**		18.163	18.143 [1]
**Bond length [Å]**	Mn-N	2.249	2.252 [3]
	N-N	1.340	1.339 [3]
	C-N	1.355	1.351 [3]
	C-C	1.386	1.361 [3]
	C-H	1.084	0.949 [3]

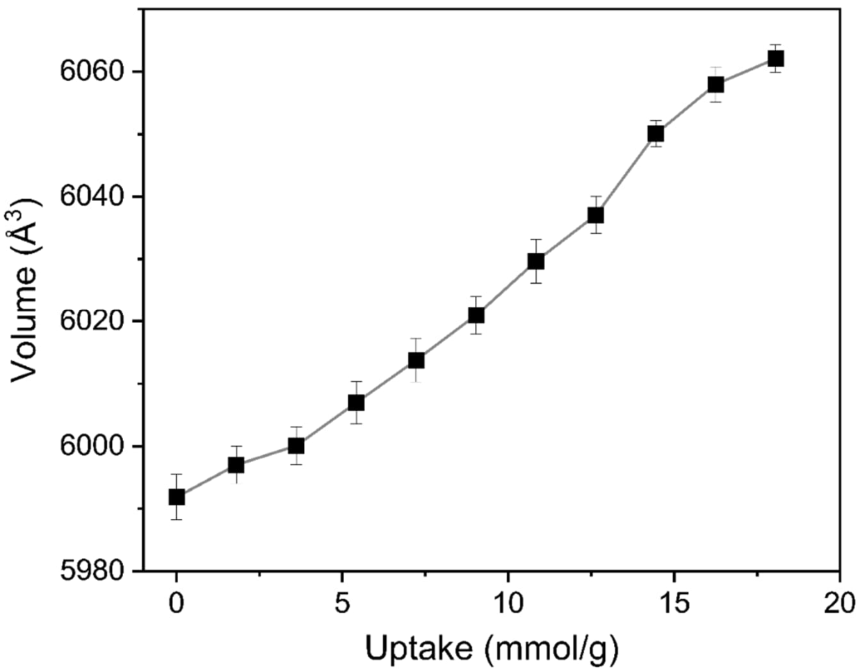

Supplementary Figure 19. **DFT volume expansion by H_2 loading.** Mn(ta)$_2$ framework volume as a function of H_2 content obtained from DFT calculations. Data points are connected by lines to guide the eye.

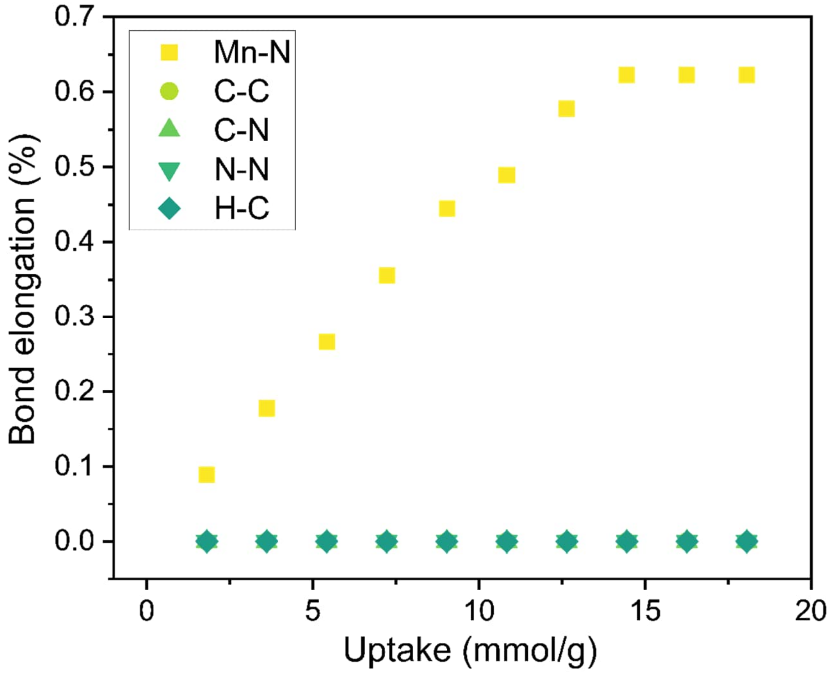

Supplementary Figure 20. **DFT bond elongation by H_2 loading.** Relative bond elongation upon loading the Mn(ta)$_2$ framework with H_2 obtained from DFT calculations. The triazole rings stay unaffected, and the volume increase is purely due to the elongation of the Mn-N bonds.

Hydrogen Isotope Separation Measurements

Thermal desorption spectroscopy (TDS)

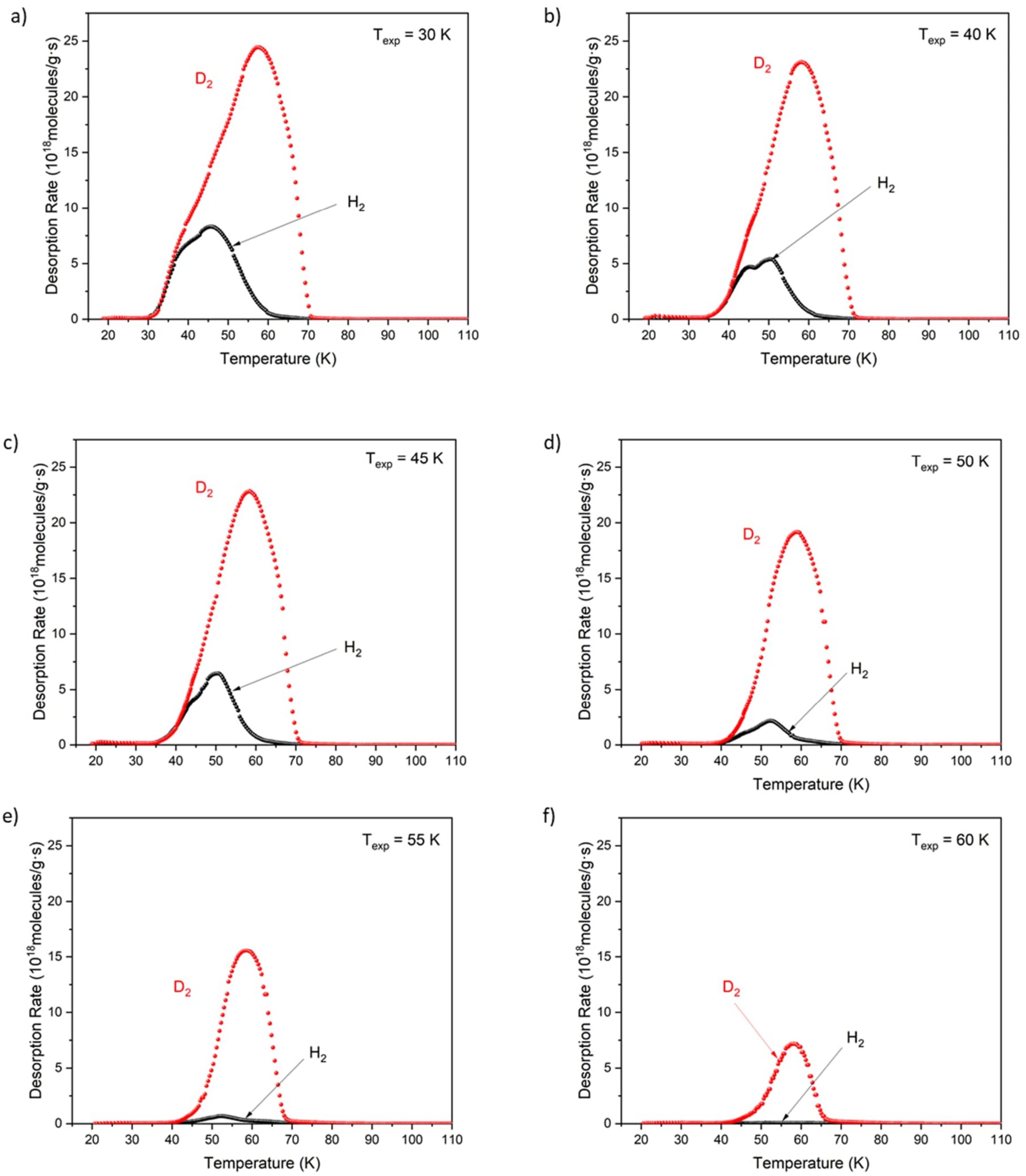

Supplementary Figure 21. **Isotope separation measured by TDS.** H_2 (black) and D_2 (red) thermal desorption spectra for 10 mbar of a 1 : 1 mixture of H_2 : D_2 for $Mn(ta)_2$ recorded over 10 min at various exposure temperatures (T_{exp}): (a) 30 K, (b) 40 K, (c) 45 K, (d) 50 K, (e) 55 K, and (f) 60 K

Supplementary Table 5. **Literature data D_2/H_2 selectivity.** Summary of experimentally measured hydrogen isotope separation performance on various porous materials.

Compound	T_{exp}(K)	Selectivity (D_2/H_2) (1:1 Mixture)	Ref.
**MFU-4 (Zn, Cl)**	40	6.9	4
	50	5.8	
	60	7.5	
**Py@COF-1**	22	9.7	5
	30	7.9	
**Cu(I)-MFU-4l**	90	7.1	6
**Fe-MOF-74**	77	2.5	7
**Co-MOF-74**	77	3.2	
**Ni-MOF-74**	77	5	
**ZIF-7**	20	-	8
**ZIF-8**	20	11	
**COF-1**	20	7	
**COF-102**	20	1	
**CPO-27-Co**	60	11.8	9
**IFP-1**	30	2.0	10
**IFP-3**	30	2.8	
**IFP-7**	77	1.5	
**IFP-4**	77	2.1	
**Zeolite 5A**	30	2.7	11
**CC3**	30	1.7	12
	50	1.8	
**6FT-RCC3**	30	2.2	
	50	3.0	
**6ET-RCC3**	30	3.9	
	50	1.8	
**Cocryst1**	30	8.0	
**MIL-53(Al)**	40	10.5	12

**MOF-74**	77	19	14
**MOF-74-IM-10**	77	26	
**MOF-74-IM-38**	40	12.5	
**5A/GE2**	77	1.07	15
**CoFA**	25	16.6	16
**ZIF-67@NH_2–SiO_2**	77	1.52	17
**FMOFCu**	25	14	18
	77	4	
**Ag(I)-ZSM-5**	77	8.7	19
**Cu(I)-ZSM-5**	100	24.9	20
**SIFSIX-1-Cu**	20	7.1	21
**SIFSIX-3-Zn**	20	50	
**SIFSIX-3-Cu**	20	3.5	
**SIFSIX-3-Ni**	20	1.9	
**HKUST-1**	20	17	
**FCTF-1-400**	20	12.8	
**STAM-1**	20	9.9	
**Cu-PYC**	20	2.2	
**CPO-27(Co)**	20	3.0	
**KAUST-7**	20	9.8	
**Ag(I)-Zeolite Y**	90	10	22
**Cu(I)Cu(II)-BTC**	30	37.9	23
**FJI-Y11**	77	1.76	24
**Ni_2Cl_2BBTA**	77	4.5	25
**Mn(ta)$_2$**	60	32.5	This work

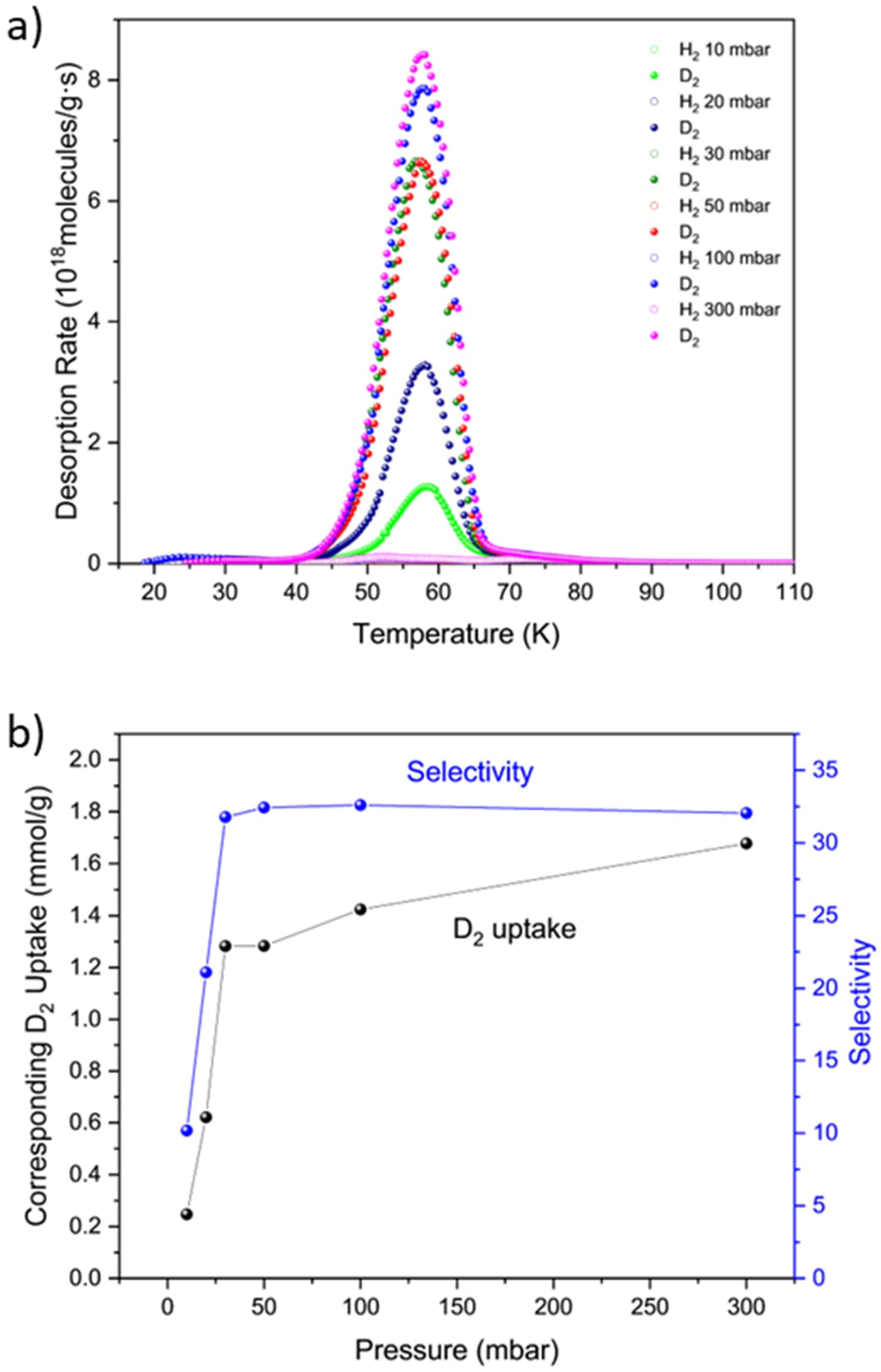

Supplementary Figure 22. **Influence of exposure pressure.** a) Hydrogen (open) and deuterium (closed) desorption spectra of a 1:1 mixture of D_2 : H_2 between 10 – 300 mbar for $Mn(ta)_2$ at an exposure temperature of 60 K for 10 min. b) The corresponding amount of adsorbed D_2 and selectivity as a function of exposure pressure obtained at T_{exp} = 60 K.

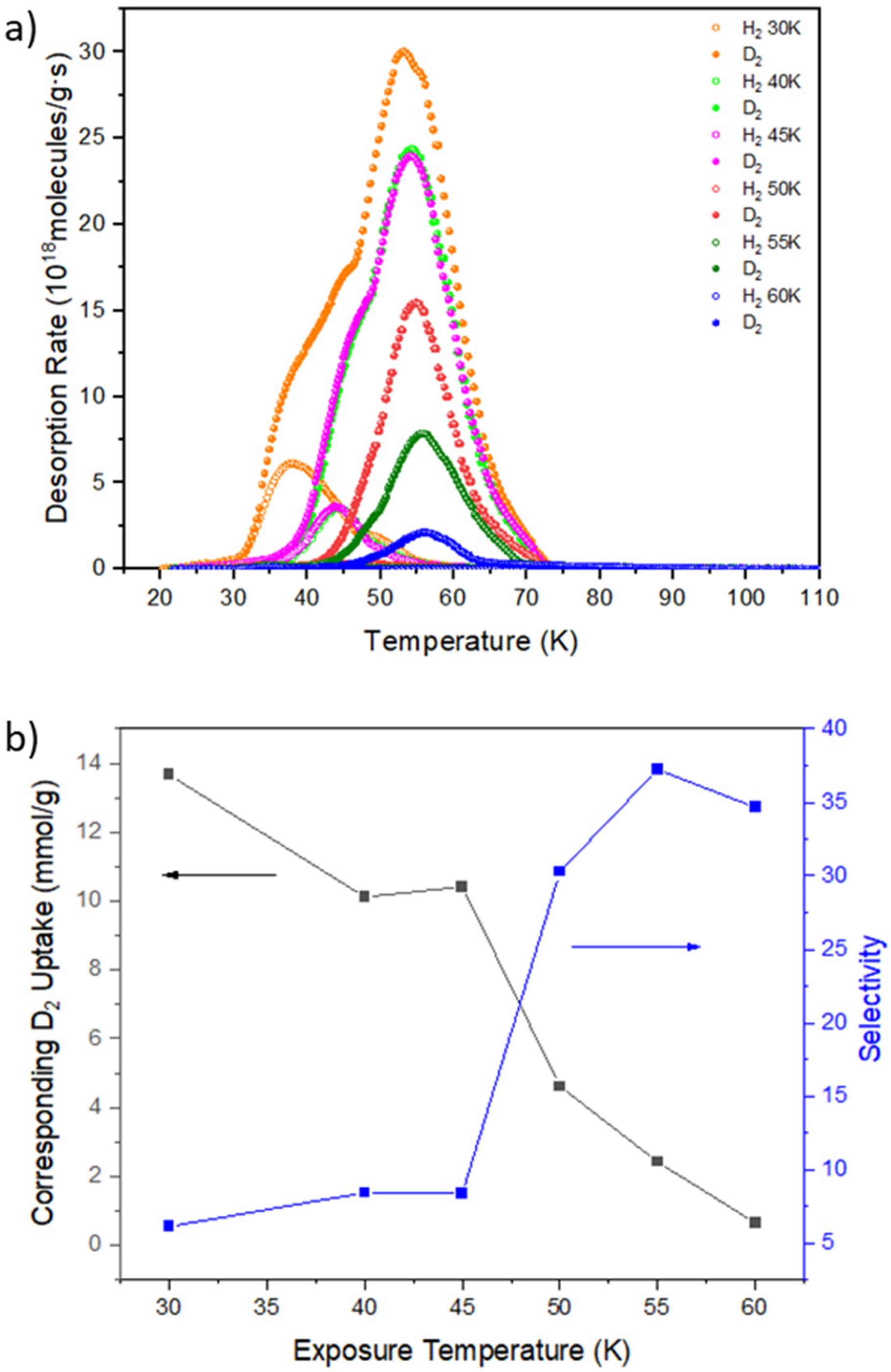

Supplementary Figure 23. **Influence of exposure temperature.** a) Hydrogen (open) and deuterium (closed) desorption spectra of a 1:1 mixture of D_2 : H_2 at 100 mbar for deuterated sample [Mn(ta-d_2)$_2$] at an exposure temperature of 60 K for 10 min. b) The corresponding amount of adsorbed D_2 and selectivity as a function of exposure temperature obtained at 100 mbar. It clearly shows that the deuterated and non-deuterated samples show similar results in hydrogen isotope separation.

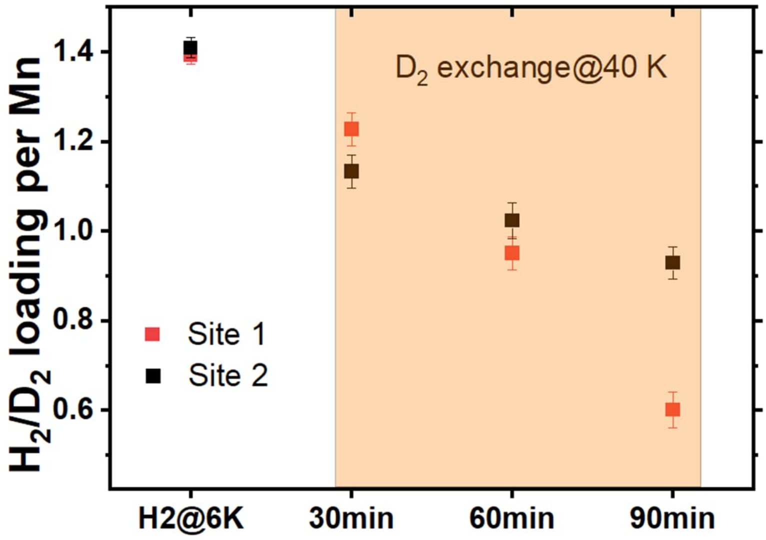

Supplementary Figure 24. **Isotope exchange at 40 K.** Isotope exchange dynamics in [Mn(ta)$_2$] monitored by neutron powder diffraction. The graph shows the decline in H_2 loading per Mn at Site 1 (red) and Site 2 (black) following D_2 introduction at 40 K. NPD data were collected initially for the H_2 loaded sample at 6 K, then at 30, 60, and 90 minutes after D_2 introduction (orange region). The more significant decrease in H_2 occupancy at Site 1 compared to Site 2 indicates preferential replacement by D_2 at the pocket sites, demonstrating their higher affinity for the heavier isotope.

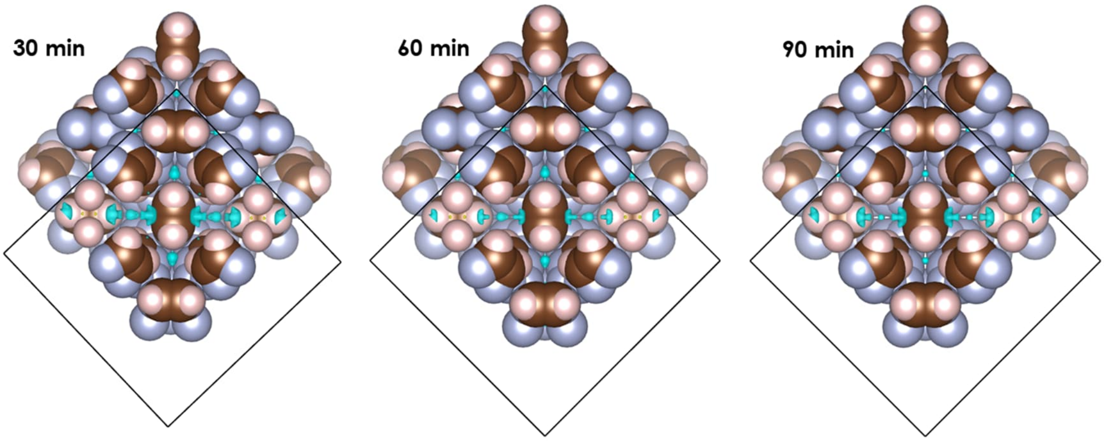

Supplementary Figure 25. **Change of scattering intensity.** Increase in scattering length density in the observed Fourier density map of [Mn(ta)$_2$] during D_2 – H_2 exchange. With increasing exposure time during the D_2 – H_2 exchange, a continuous increase in the scattering length density was observed.

Supplementary References

1. Gándara, F., Uribe-Romo, F. J., Britt, D. K., Furukawa, H., Lei, L., Cheng, R., Duan, X., O'Keeffe, M., Yaghi, O. M. Metal-triazolates and their structural elucidation by the charge-flipping method. *Chem. Eur. J.* **18**, 10595–10601 (2012).
2. Li, J. R., Kuppler, R. J., Zhou, H. C. Selective gas adsorption and separation in metal-organic frameworks. *Chem. Soc. Rev.* **38**, 1477–1504 (2009).
3. He, C-T., Ye, Z-M., Xu, Y-T., Zhou, D-D., Zhou, H-L., Chen, D., Zhang, J-P., Chen, X-M. Hyperfine adjustment of flexible pore-surface pockets enables smart recognition of gas size and quadrupole moment. *Chem. Sci.* **8**, 7560–7565 (2017).
4. Teufel, J., Oh, H., Hirscher, M., Wahiduzzaman, M., Zhechkov, L., Kuc, A., Heine, T., Denysenko, D., Volkmer, D. MFU-4 - A metal-organic framework for highly effective H_2/D_2 separation. *Adv. Mater.* **25**, 635–639 (2013).
5. Oh, H., Kalidindi, S. B., Um, Y., Bureekaew, S., Schmid, R., Fischer, R. A., Hirscher, M. A cryogenically flexible covalent organic framework for efficient hydrogen isotope separation by quantum sieving. *Angew. Chem. Int. Ed.* **52**, 13219-13222 (2013).
6. Weinrauch, I., Savchenko, I., Denysenko, D., Souliou, S. M., Kim, H. H., Le Tacon, M., Daemen, L. L., Cheng, Y., Mavrandonakis, A., Ramirez-Cuesta, A. J., Volkmer, D., Schütz, G., Hirscher, M., Heine, T. Capture of heavy hydrogen isotopes in a metal-organic framework with active Cu(I) sites. *Nat. Commun.* **8**, 14496 (2017).
7. FitzGerald, S. A., Pierce, C. J., Rowsell, J. L., Bloch, E. D., Mason, J. A. Highly selective quantum sieving of D_2 from H_2 by a metal–organic framework as determined by gas manometry and infrared spectroscopy. *J. Am. Chem. Soc.* **135**, 9458-9464 (2013).
8. Oh, H., Park, K. S., Kalidindi, S. B., Fischer, R. A., Hirscher, M. Quantum cryo-sieving for hydrogen isotope separation in microporous frameworks: an experimental study on the correlation between effective quantum sieving and pore size. *J. Mater. Chem. A* **1**, 3244-3248 (2013).
9. Oh, H., Savchenko, I., Mavrandonakis, A., Heine, T., Hirscher, M. Highly effective hydrogen isotope separation in nanoporous metal-organic frameworks with open metal sites: Direct measurement and theoretical analysis. *ACS Nano* **8**, 761–770 (2014).
10. Mondal, S. S., Kreuzer, A., Behrens, K., Schütz, G., Holdt, H. J., Hirscher, M. Systematic experimental study on quantum sieving of hydrogen isotopes in metal-amide-imidazolate frameworks with narrow 1-D channels. *ChemPhysChem* **20**, 1311 (2019).
11. Xiong, R., Xicohtencatl, R. B., Zhang, L., Li, P., Yao, Y., Sang, G., Chen, C., Tang, T., Luo, D., Hirscher, M. Thermodynamics, kinetics and selectivity of H_2 and D_2 on zeolite 5A below 77K. *Micropor. Mesopor. Mat.* **264**, 22-27 (2018).
12. Liu, M., L. Zhang, L., M. A. Little, M. A., V. Kapil, V., M. Ceriotti, M., S. Yang, S., L. Ding, L., D. L. Holden, D. L., R. Balderas-Xicohténcatl, R., D. He, D., R. Clowes, R., S. Y. Chong, S. Y., G. Schütz, G., L. Chen, L., M. Hirscher, M., A. I. Cooper, A. I. Barely porous organic cages for hydrogen isotope separation. *Science* **366**, 613-620 (2019).

13. Kim, J. Y., Zhang, L., Balderas-Xicohténcatl, R., Park, J., Hirscher, M., Moon, H. R., Oh, H. Selective hydrogen isotope separation via breathing transition in MIL-53 (Al). *J. Am. Chem. Soc.* **139**, 17743-17746 (2017).
14. Kim, J. Y., Balderas-Xicohténcatl, R., Zhang, L., Kang, S. G., Hirscher, M., Oh, H., Moon, H. R. Exploiting diffusion barrier and chemical affinity of metal–organic frameworks for efficient hydrogen isotope separation. *J. Am. Chem. Soc.* **139**, 15135-15141 (2017).
15. Sun, N., Li, P.-L., Wen, M., Song, J.-F., Zhang, Z., Yang, W.-B., Zhou, Y.-L., Luo, D.-L., Zhang, Q.-P. Insights into heat management of hydrogen adsorption for improved hydrogen isotope separation of porous materials. *J. Mater. Sci. Technol.* **76**, 200-206 (2021).
16. Muhammad, R., Jee, S., Jung, M., Park, J., Kang, S. G., Choi, K. M., Oh, H. Exploiting the specific isotope-selective adsorption of metal–organic framework for hydrogen isotope separation. *J. Am. Chem. Soc.* **143**, 8232-8236 (2021).
17. Chen, X., Liu, M., Zhang, L., Zhou, Y., Ping, E., Ding, C. New gas chromatographic packing ZIF-67@ NH_2–SiO_2 for separation of hydrogen isotope H_2/D_2. *Int. J. Hydrogen Energy* **46**, 13029-13037 (2021).
18. Zhang, L., Jee, S., Park, J., Jung, M., Wallacher, D., Franz, A., Lee, W., Yoon, M., Choi, K., Hirscher, M., Oh, H. Exploiting dynamic opening of apertures in a partially fluorinated MOF for enhancing H_2 desorption temperature and isotope separation. *J. Am. Chem. Soc.* **141**, 19850–19858 (2019).
19. Xiong, R., Chen, J., Zhang, L., Li, P., Yan, X., Song, Y., Luo, W., Tang, T., Sang, G., Hirscher, M. Hydrogen isotopes separation in Ag (I) exchanged ZSM-5 zeolite through strong chemical affinity quantum sieving. *Micropor. Mesopor. Mat.* **313**, 110820 (2021).
20. Xiong, R., Zhang, L., Li, P., Luo, W., Tang, T., Ao, B., Sang, G., Chen, C., Yan, X., Chen, J. Highly effective hydrogen isotope separation through dihydrogen bond on Cu(I)-exchanged zeolites well above liquid nitrogen temperature. *Chem. Eng. J.* **391**, 123485 (2020).
21. Han, G., Gong, Y., Huang, H., Cao, D., Chen, X., Liu, D., Zhong, C. Screening of metal–organic frameworks for highly effective hydrogen isotope separation by quantum sieving. *ACS Appl. Mater. Interfaces* **10**, 32128-32132 (2018).
22. Zhang, L., Wulf, T., Baum, F., Schmidt, W., Heine, T., Hirscher, M. Chemical affinity of Ag-exchanged zeolites for efficient hydrogen isotope separation. *Inorg. Chem.* **61**, 9413–9420 (2022).
23. Hu, X., Ding, F., Xiong, R., An, Y., Feng, X., Song, J., Zhou, L., Li, P., Chen, C. Highly Effective H_2/D_2 Separation within the stable Cu(I)Cu(II)-BTC: The effect of Cu(I) structure on quantum sieving. *ACS Appl. Mater. Interfaces* **15**, 3941-3952 (2023).
24. Si, Y., He, X., Jiang, J., Duan, Z., Wang, W., Yuan, D. Highly effective H_2/D_2 separation in a stable Cu-based metal-organic framework. *Nano Research* **14**, 518-525 (2021).
25. Li, X., Wang, X., Li, M., Luo, J., An, Y., Li, P., Song, J., Chen, C., Feng, X., Wang, S. Highly selective adsorption of D_2 from hydrogen isotopes mixture in a robust metal bistriazolate framework with open metal sites. *Int. J. Hydrogen Energy* **45**, 21547-21554 (2020).
